# Principles of Botany as Exemplified in the Cryptogamia

**Published:** 1853-09

**Authors:** 


*Principles of Botany as exemplified in the Cryptogamia.* By HARLAND COULTAS.

Here is an illustration of the phenomena of cell-life, drawn from the lowest order of vegetation. He traces vegetation from a single row of cells, (as in the bread mould,) up to its full development in the complicated arrangement of cell-tissue in the forest tree. Our author confines himself to cell-development in this little work, leaving the circulatory function untouched. The molecular theory of Dr. Backus, mentioned above, finds its analogue in vegetable circulation. The presence of aggregation, and disintegration, of capillary tubes, of heat, moisture, and the different density, and therefore pressure of the earth about the roots, and the air pressure on the trunk and leaves, furnish all the necessary elements of unity. The bursting of cells from too abundant sap, is the result, it would seem, of vegetable congestion.

For sale at PHINNEY'S. S. B. H.

